# Repetitive Traumatic Brain Injury Causes Neuroinflammation before Tau Pathology in Adolescent P301S Mice

**DOI:** 10.3390/ijms22020907

**Published:** 2021-01-18

**Authors:** Saef Izzy, Alexander Brown-Whalen, Taha Yahya, Aliyah Sarro-Schwartz, Gina Jin, Joon Yong Chung, Sevda Lule, Liza M. Morsett, Ali Alquraini, Limin Wu, Suzanne E. Hickman, Michael J. Whalen, Joseph El Khoury

**Affiliations:** 1 Department of Neurology, Brigham and Women’s Hospital, Boston, MA 02115, USA; sizzy@bwh.harvard.edu (S.I.); tyahya@bwh.harvard.edu (T.Y.); aliyahss@alumni.stanford.edu (A.S.-S.); 2 Center for Immunology and Inflammatory Diseases, Massachusetts General Hospital, 149 13th Street, Charlestown, MA 02119, USA; ABROWN-WHALEN@PARTNERS.ORG (A.B.-W.); LMORSETT@mgh.harvard.edu (L.M.M.); AALQURAINI@mgh.harvard.edu (A.A.); SHICKMAN@mgh.harvard.edu (S.E.H.); 3 Harvard Medical School, Boston, MA 02115, USA; MWHALEN@mgh.harvard.edu; 4 Department of Pediatrics, Massachusetts General Hospital, Boston, MA 02114, USA; gina.jin@med.uvm.edu (G.J.); jchung8@student.nymc.edu (J.Y.C.); SLULE@mgh.harvard.edu (S.L.); LWU11@mgh.harvard.edu (L.W.); 5 Faculty of Clinical Pharmacy, Al Baha University, Al Baha 65779, Saudi Arabia

**Keywords:** concussion, tau, adolescents, traumatic brain injury, CTE, microglia

## Abstract

Repetitive closed head injury (rCHI) is commonly encountered in young athletes engaged in contact and collision sports. Traumatic brain injury (TBI) including rCHI has been reported to be an important risk factor for several tauopathies in studies of adult humans and animals. However, the link between rCHI and the progression of tau pathology in adolescents remains to be elucidated. We evaluated whether rCHI can trigger the initial acceleration of pathological tau in adolescent mice and impact the long-term outcomes post-injury. To this end, we subjected adolescent transgenic mice expressing the P301S tau mutation to mild rCHI and assessed tau hyperphosphorylation, tangle formation, markers of neuroinflammation, and behavioral deficits at 40 days post rCHI. We report that rCHI did not accelerate tau pathology and did not worsen behavioral outcomes compared to control mice. However, rCHI induced cortical and hippocampal microgliosis and corpus callosum astrocytosis in P301S mice by 40 days post-injury. In contrast, we did not find significant microgliosis or astrocytosis after rCHI in age-matched WT mice or sham-injured P301S mice. Our data suggest that neuroinflammation precedes the development of Tau pathology in this rCHI model of adolescent repetitive mild TBI.

## 1. Introduction

Concussions continue to be a prominent public health concern, with an estimated 1.6–3.8 million cases annually in the United States [1]. Younger individuals, ages 14–19, have the highest rates of concussion and nearly all athletic activities carry some risk of concussive injury [1,2]. The long-term cognitive outcomes of repetitive concussive head injuries remain disputed, but there have been numerous epidemiological and neuropathological reports of an association with cognitive decline and neurodegenerative disease including chronic traumatic encephalopathy (CTE) [3,4,5,6]. Concussion in young athletes may increase the risk of chronic cognitive dysfunction compared to their adult counterparts [7]. Such concerns, coupled with the popularity of youth participation in contact sports, have made adolescent traumatic brain injury (TBI) a central focus of public attention [3,4,5,6].

CTE is a tauopathy characterized by the deposition of hyperphosphorylated tau (p-tau) protein as neurofibrillary tangles (NFTs), astrocytic tangles (ATs), and neurites in striking clusters around small blood vessels of the cortex, typically at the sulcal depths [8,9]. CTE has received considerable public attention due to recent studies showing CTE in the brains of long-term survivors of TBI, adult athletes, and war veterans [10,11]. In addition to adults, recent studies found CTE-like pathology including astrocytosis, axonopathy, and neuroinflammation in postmortem brains from teenage athletes [6,12]. Moreover, p-tau and NFT pathology have been found in the brains of adolescents within hours following injury [13,14,15]. Nevertheless, the relationship between TBI and progressive tauopathies such as CTE remains disputed [16,17]. Several pre-clinical TBI studies have shown increased tau phosphorylation in mouse models of tauopathy at acute and subacute time points [18,19] Many others showed evidence of gliosis and white matter degeneration, which recapitulate key CTE features [20,21,22]. These studies sought to explore the TBI-CTE link with adult 4–18 months old mice [18,23,24], using diffuse closed head injury and focal contusion models of TBI [18,24]. Recent longitudinal studies using adult transgenic mice expressing human tau and rCHI models showed evidence of white matter disease and cognitive and motor dysfunction up to one-year post-injury, without showing a difference in p-tau deposition between sham and injured mice [25]. Despite the prevalence of adolescent concussions and the recently reported risk of CTE in this population, the impact of rCHI on the progression of tau pathobiology in adolescent mice has not been fully explored. One repetitive mild TBI study in adolescent tauopathy mice showed increased tau (pS422 immunolabeling) in the visual system but not in the cortex [26]. Human TBI and experimental studies have also shown persistent microglial activation and tau pathology after rCHI [27,28,29,30], which coincide with axonal abnormality and chronic neuronal degeneration [31,32,33]. These findings suggest a potential role for a persistent neuroinflammatory response in the development of TBI-induced neuronal injury and CTE. This is supported by findings in conditions other than TBI. Indeed, chronic neuroinflammation resulting from the interactions of microglial scavenger and Toll-like receptors with noxious stimuli such as β-amyloid or lipopolysaccharide leads to the generation of neurotoxic substances and subsequently contribute to the pathophysiology of neurodegenerative disorders [34,35,36,37,38,39]. At present, the impact of both rCHI and p-tau pathology on neuroinflammation and behavioral outcomes in adolescent mice remain poorly understood.

To address this knowledge gap, we explored the relationship between adolescent concussion, neuroinflammation, and tauopathy using adolescent P301S transgenic mice that harbor a mutant form of the human microtubule-associated protein tau (MAPT) and exhibit similar tau pathology to human tauopathies [40,41]. We found that at around 3 months of age, the time these mice have reached maturity [42], rCHI induced microgliosis and astrocytosis in P301S mice without accelerating tau pathology or inducing behavioral deficits.

## 2. Results

### 2.1. Repetitive Closed-Head Injury (rCHI) Did Not Alter Tau Pathology in Adolescent P301S Mice 40 Days Post Injury

**Figure 1:**
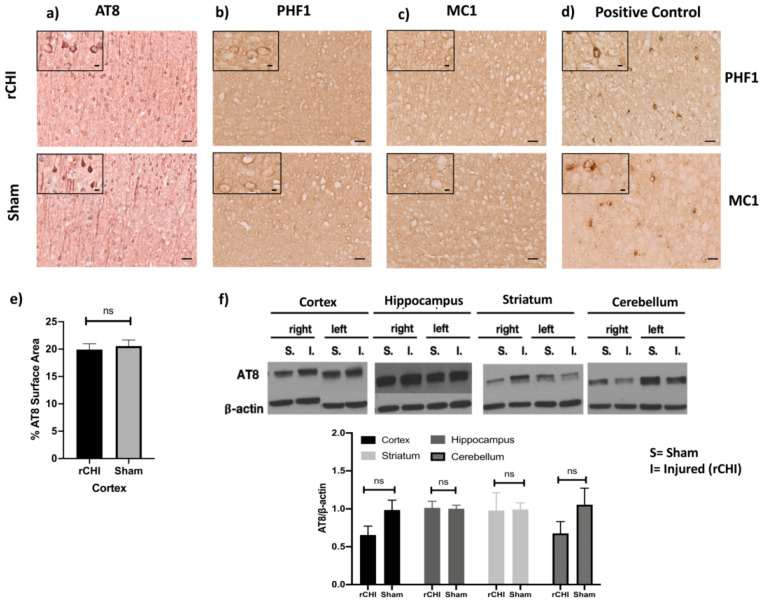
Repetitive closed-head injury (rCHI) did not alter tau pathology in adolescent P301S mice 40 days post-injury. Representative immunohistochemistry (IHC) of (**a**) AT-8 which shows no increase in tau phosphorylation in cortical neuronal cell bodies in rCHI vs. sham P301S mice at 40 days post-injury. (**b**,**c**) Representative IHC of (**b**) PHF1, (**c**) MCI of the cortex of P301S rCHI and sham mice show a paucity of tau staining with only a few cortical neuronal cell bodies in both sham and injured P301S mice at 40 days post-injury. (**d**) widespread tau staining (both PHF1and MC1) in 270-day old naïve P301S mice. (**e**) Bar graphs show that no significant difference in cortical AT-8 surface area between rCHI and sham P301S mice (*n* = 4/group, *p* = 0.67). (**f**) Bar graphs show that no change in AT-8 in brain homogenates of P301S at 40 days after rCHI vs. sham across all groups (*n* = 5/group, *p* = 0.09 for cortex, *p* = 0.91 for hippocampus, *p* = 0.96 for striatum, *p* = 0.91 for cerebellum). Nonspecific Western blot bands were cropped for clarity in panels. Statistical significance is denoted as follows: ns (non-significant) *p* > 0.05. Scale bars are 50 μm, and 10 μm for photomicrographs on increasing magnification.

To assess whether mild repetitive TBI during adolescence, when many athletes become exposed to contact and collision sports, accelerates tauopathy we used P301S and WT adolescent mice aged 38 ± 3 days. We used immunohistochemistry to investigate the pattern of tauopathy in the brains of these mice, 40 days after receiving three repetitive injuries. This time point was used because at this age (~78 days old) the mice have reached adulthood and are considered mature [42]. We used AT8 as a marker of phospho-tau, which targets the phospho-tau epitope at serine residue (ser202/thr205). AT8 staining was detected in the cell body and apical dendrites of neurons in the cortex, but not in the hippocampus, of both sham and injured groups of P301S mice (Figure 1a). rCHI did not significantly increase cortical tau phosphorylation in injured vs. sham P301S mice as measured by percentage surface area stained by AT8 (Figure 1e). We also assessed the effects of rCHI on the development of late-stage neurofibrillary-like pathology using PHF1, which recognizes phospho-tau epitopes at Serine 396 and Serine 404 residues. The immunolabeling of PHF1 showed a paucity of staining with only a few cortical neuronal cell bodies staining at 40 days post-injury in both sham and injured groups. In contrast, 270-day-old naïve uninjured P301S mice showed widespread staining and were used as a positive control (Figure 1b,d). The cortex and hippocampus were also stained with MC1 to evaluate for toxic tau conformational species, but there was insufficient positive staining for surface area percentage quantification at 80 ± 3 days of age in both sham and injured compared to a marked increase in MC1 staining in the 270-day old naïve uninjured positive control P301S/5XFAD mice (Figure 1c,d). In support of these findings, Western blot analysis of brain homogenates from cortical, hippocampal, striatal, and cerebellar tissue using the AT8 antibody showed no significant differences in relative composition between sham and injured groups (Figure 1f).

### 2.2. Repetitive Closed-Head Injury (rCHI) Induced Microgliosis in Adolescent P301S but Not WT Mice at 40 Days Post Injury

**Figure 2:**
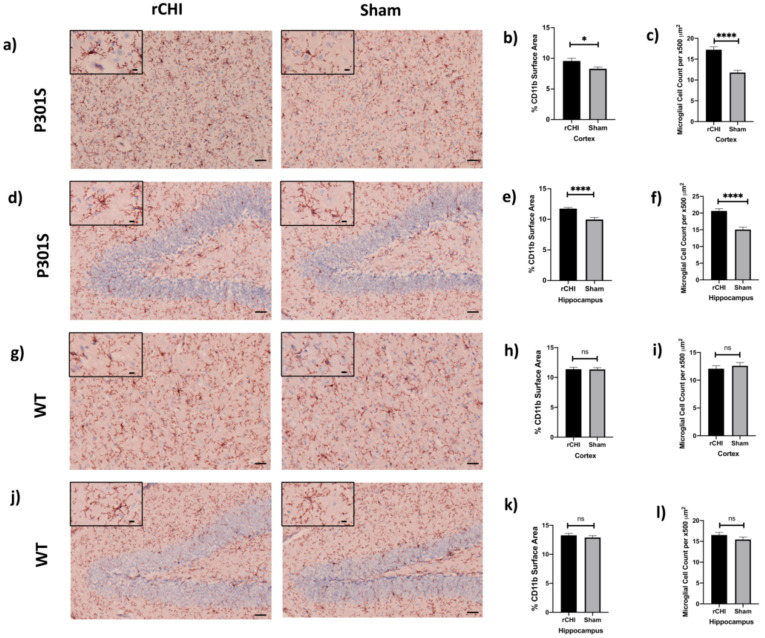
Repetitive closed-head injury (rCHI) induced microgliosis in adolescent P301S but not WT mice at 40 days post-injury. Representative IHC of CD11b of (**a**) cortex and (**d**) hippocampus of rCHI and sham P301S mice at 40 days post-injury. Bar graphs show increase in percent area of CD11b staining in (**b**) cortex and (**e**) hippocampus in rCHI vs. sham P301S mice (*n* = 4/group, *p* = 0.02, *p* < 0.0001, respectively). Representative IHC of CD11b of (**g**) cortex and (**j**) hippocampus of rCHI and sham WT mice at 40 days post-injury. Bar graphs show no significant difference in percent area of CD11b between rCHI and sham WT animals in both (**h**) cortex and (**k**) hippocampus (*n* = 4/group, *p* = 0.92, *p* = 0.42, respectively). Manual quantification of CD11b positive cells shows significant increase in CC11b in (**c**) the cortex and (**f**) the hippocampus of rCHI compared to sham P301S mice (*n* = 3/group, *p* < 0.0001, *p* < 0.0001, respectively). Manual quantification of CD11b positive cells in (**i**) cortex and (**l**) hippocampus of rCHI and sham WT mice showed no significant differences between the groups (*n* = 4/group, *p* = 0.51, *p* = 0.21, respectively). Statistical significance is denoted as follows: ns (non-significant) *p* > 0.05, * *p* ≤ 0.05, **** *p* ≤ 0.0001. Scale bars are 50 μm, and 10 μm for photomicrographs on increasing magnification.

To assess the extent of microgliosis in response to rCHI, immunostaining with CD11b antibody was used and the cortex and hippocampus of injured P301S and WT mice were analyzed and compared to shams at 40 days post-injury (Figure 2a–l). Immunostained sections of the cortex and hippocampus showed a significant increase in the percent of CD11b+ surface area in injured P301S mice compared to sham P301S mice (Figure 2b,e). The increased surface area staining corresponded to a significant increase in the number of CD11b+ cells suggesting either recruitment or proliferation of microglia. In contrast, we found no significant difference in the percentage of CD11b+surface area or the number of CD11b+ cells in WT injured mice compared to shams (Figure 2h,k). In line with these results, a significant increase was found in the manual count of CD11b+ cells in both cortex and hippocampus of P301S mice (Figure 2c,f). However, there was no change in the manual count of CD11b+ cells in both cortex and hippocampus of WT mice (Figure 2i,l).

### 2.3. Repetitive Closed-Head Injury (rCHI) Increased Astrocytosis in Adolescent P301S but Not in WT Mice at 40 Days Post Injury

**Figure 3:**
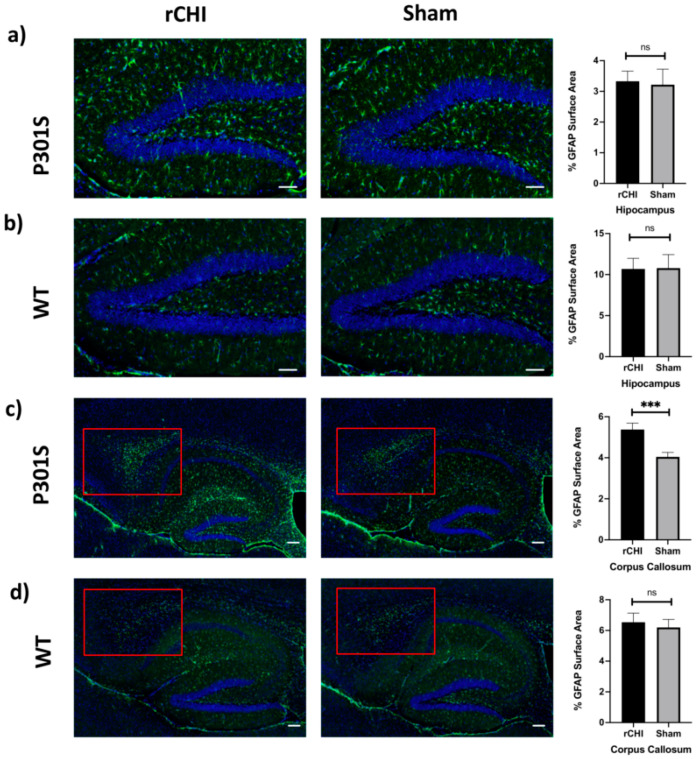
Repetitive closed-head injury (rCHI) increased astrocytosis in adolescent P301S but not in WT mice at 40 days post-injury. (**a**,**b**) Representative IHC of GFAP staining in the hippocampus of rCHI and sham P301S (**a**) and WT mice (**b**) at 40 days post-injury. Percent area of GFAP staining was not significantly different in the hippocampus of rCHI compared to sham in both P301S and WT mice (*n* = 4/group, *p* = 0.85, *p* = 0.96, respectively). (**c**,**d**) Representative IHC of GFAP in the corpus callosum of rCHI and sham P301S (**c**) and WT (**d**) mice at 40 days post-injury. Percent area of GFAP staining was significantly increased in the corpus callosum of rCHI vs. sham P301S mice (**c**), but was not significantly different in in the corpus callosum of rCHI vs. sham WT mice at 40 days post-injury (**d**) (*n* = 4/group, *p* = 0.0009, *p* = 0.67 respectively). Red box illustrates the increased astrocytosis in the corpus callosum of rCHI compared to sham in P301S, but not in WT, mice groups. Statistical significance is denoted as follows: ns (non-significant) *p* > 0.05, *** *p* ≤ 0.001. Scale bars are 50 μm, and 20 μm for photomicrographs on increasing magnification.

To assess the extent of astrocytosis in response to rCHI, we used immunofluorescence staining for GFAP and analyzed the hippocampus and corpus callosum of injured P301S mice compared to shams at 40 days post-injury (Figure 3). GFAP-positive astrocytes are usually concentrated most in the hippocampus and they were less detected in the cortical region [43]. Immunostained sections of the hippocampus did not show a significant difference in the percent surface area stained for GFAP (Figure 3a,b). Interestingly, there was a significant increase in the percent of the GFAP+ surface area in the corpus callosum of adolescent P301S mice at 40 days post rCHI versus shams (Figure 3c,d). The percent surface area e for GFAP staining was not significantly different in both hippocampus and corpus callosum of adolescent WT mice at 40 days post rCHI versus shams (Figure 3c,d).

### 2.4. No Behavioral Deficits Were Detected 40 Days after Repetitive Closed-Head Injury (rCHI) in Adolescent P301S and WT Mice

**Figure 4:**
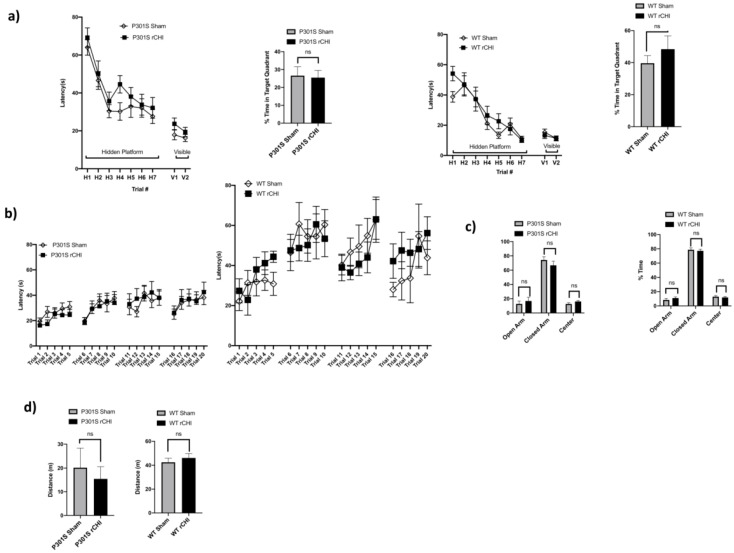
No behavioral deficits were detected 40 days after repetitive closed-head injury (rCHI) in adolescent P301S and WT mice. (**a**) There was no significant difference in Morris water maze (MWM) and Probe trial testing between rCHI vs. sham P301S mice (*n* = 14/group, *p* = 0.16, *p* = 0.87 respectively) or rCHI vs. sham WT mice (*n* = 6/group, *p* = 0.61, *p* = 0.38 respectively). (**b**) There were no differences in the rotarod testing between rCHI vs. sham P301S mice (*n* = 14/group, *p* = 0.95), or rCHI vs. sham WT mice (*n* = 6/group, *p* = 0.88). (**c**) There were no differences in the % time spent in the open, closed, and center arm of the plus maze between rCHI vs. sham P301S mice (*n* = 14/group, *p* = 0.53, 0.32, and 0.15, respectively), or rCHI vs. sham WT mice (*n* = 6/group, *p* = 0.38, 0.68, 0.62, respectively). (**d**) No differences were observed in open-field tests among rCHI vs. sham P301S mice (*n* = 10/group, *p* = 0.63, or rCHI vs. sham WT mice (*n* = 6/group, *p* = 0.48). Statistical significance is denoted as follows: ns (non-significant) *p* > 0.05. Data are mean ± SEM. SEM, standard error of the mean.

To investigate spatial memory and learning deficits post rCHI, we used the Morris water maze (MWM) and probe trial testing. We did not find significant differences between injured adolescent P301S and sham controls. Similarly, there were no significant differences in hidden platform trials and probe trials in injured vs. sham WT mice (Figure 4a). Rotarod testing for motor activity revealed no significant differences between injured adolescent P301S vs. sham controls groups or between adolescent WT mice injured compared to sham groups (Figure 4b). We also tested the performance of the mice in the elevated Plus Maze to assess for underlying anxiety and found no significant differences between the injured adolescent P301S and sham controls groups. Specifically, there were no differences in the percentage of time spent in the open arm, closed arm, and center across both the P301S and sham control groups. Similarly, no differences were observed among adolescent WT injured vs. sham controls (Figure 4c). Open-field testing was used to assess activity levels and willingness to explore also showed no significant differences between sham and injured adolescent P301S groups and no differences were observed among adolescent WT mice injured vs. sham (Figure 4d).

## 3. Discussion

As participation in youth sports has risen over the past two decades, concussion and its association with long-term neurodegenerative risk have become a topic of significant public concern, especially since chronic traumatic encephalopathy (CTE) pathology, a form of tauopathy, has been reported in brains of deceased teenage athletes [6,12]. Although not all individuals exposed to repetitive closed-head injury will develop CTE [44], several epidemiological and preclinical studies suggest that TBI is a risk factor for dementia and tauopathies in adults [45,46,47] and preclinical studies showed increased tau phosphorylation at acute and subacute timepoints in aged mice [18]. However, the link between rCHI and progression of Tau neuropathology has not been well studied in adolescent human brains or animal models. In this study, we aimed to address this knowledge gap using adolescent P301S mice which harbor a 1N4R human tau gene mutated at the 301 site (proline to serine) rendering the tau protein more prone to aggregation [40,41]. These mice were injured at 38–40 days old when phosphorylated tau and tangles are not yet present in the hippocampus or cortex, and to model the age at which young adolescent humans might sustain repetitive head injuries. We then analyzed these mice 40 days post-injury, because at this age (78–80 days old) the mice have become adults and are considered mature [42].

We found that a three-hit daily repetitive concussive injury in adolescent P301S, but not wild type mice resulted in neuroinflammation at 40 days post-injury, defined by cortical and hippocampal microgliosis and corpus callosum astrocytosis. However, there was no observed change in phosphorylated or tangled tau in brain tissues at 40 days post-injury as assessed by immunostaining with several anti-tau antibodies (AT8, PHF1, and MC1) which recognize three different pathological p-tau epitopes. Moreover, no differences were observed in cognitive or motor function between sham and injured WT and P301S mice up to 40 days post-injury.

There are several potential explanations for the lack of behavioral deficits or tau pathology in injured P310S mice. First, the duration of follow-up may not be long enough to observe significant tauopathy. Indeed, in one rCHI study using a 42-impact closed head injury of adult tau and WT mice, accelerated neuroinflammation and tau pathology were observed at a later time point (3 months) post-injury in multiple brain regions of adult tau compared to WT mice [48]. Second, it is possible that our rCHI model was not severe enough to trigger excessive tau phosphorylation. In line with our findings, previous studies showed that repetitive mild TBI models using 4 and 12 injuries in adolescent P301S mice increased tau (pS422 immunolabeling) in the visual system, but not in the cortex or hippocampus [26]. These findings suggest that the acute or subacute increase in tau phosphorylation seen in some rCHI studies may reflect a neuroinflammation-independent neuronal reaction to trauma that resolves with time [49,50,51]. In aggregate, these findings suggest that the age of onset of TBI as well as the post-injury interval at which the animals are analyzed may affect whether tau pathology is observed in these animals.

In addition to rCHI, severe TBI models such as controlled cortical impact (CCI), a model of contusion TBI, accelerated tau pathology in different brain regions of adult P301S mice that increased over time compared with sham injured mice [47]. Unlike contusion TBI, the formation of tau misfolded aggregates may take a longer time to develop after rCHI which exhibits a distinct immune response from the one that accompanies CCI and cerebral contusion [24,52]. It is also possible that the age at which the mice were injured is a contributing factor. Indeed, rCHI led to acute and subacute increases in tau phosphorylation in adult tau transgenic mice [48]. Third, the genetic background of inbred mice with the mutant human tau gene might not be permissive to trigger progressive tauopathy after mild TBI [19].

One of the key aspects of long-term sequelae of TBI, including CTE, is the development of behavioral symptoms including increased impulsivity, depression, and cognitive deficits. We did not find any significant changes in behavioral outcomes at 40 days post rCHI in wild type or P310S mice compared to sham controls. This is not surprising since such changes are usually observed following the development of Tau pathology [53].

**Figure 5:**
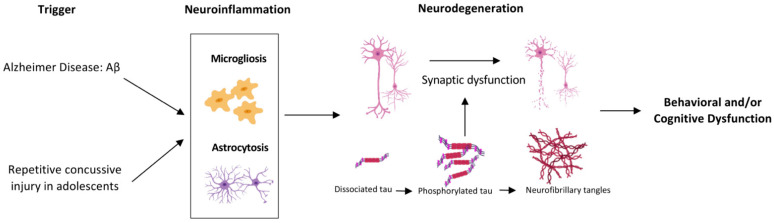
Proposed parallel mechanism between Alzheimer’s disease and Repetitive Concussive Injury. Our observation of increased microglia and astrocytes post rCHI in P301S mice in the absence of tau pathology and behavioral dysfunction or neuronal loss points to a potential parallel mechanism to that observed in Alzheimer’s disease where the inflammatory response develops likely as a result of Amyloid β deposition and thereafter contributes to the development of tau pathology and cognitive dysfunction. It is possible that recurrent concussion, similar to Amyloid β deposition is the initial trigger for a similar cascade of events. Created with BioRender.com.

Microglial and astrocyte activation has been reported at acute and chronic time points post-TBI, as these cells play a pivotal role in the brain’s response to injury [34,54,55]. Previous studies have also shown evidence of microglial cell activation in adult humanized Tau mice (hTau) at acute and chronic time points post rCHI [19]. Our study extends these findings to injured P301S adolescent mice and shows significant microgliosis in the cortex and hippocampus, two months earlier than the onset of gliosis typically observed in uninjured naïve P301S mice [56]. Neuroinflammation is known to contribute to the pathogenesis of neurodegenerative disorders including AD and CTE [34,54,57,58]. In both clinical and preclinical AD studies, microglial activation has been shown to develop after amyloid deposition but before tau aggregation, neuronal loss, and behavioral dysfunction [53,57,59]. This led us to propose that β-amyloid induced microglial activation results in dysregulation in their housekeeping functions and neuroinflammatory response, resulting in activation of pathways that contribute to tau phosphorylation and aggregation as well as the production of reactive oxygen and nitrogen species and other neurotoxins that cause neuronal damage [34]. Our observation of increased microgliosis and astrocytosis post rCHI in P301S mice in the absence of tau pathology and behavioral dysfunction or neuronal loss points to a potential parallel mechanism to that observed in AD where the inflammatory response develops likely as a result of amyloid-β deposition and thereafter contributes to the development of tau pathology and cognitive dysfunction [53]. It is possible that recurrent concussion, similar to Amyloid β deposition in AD, is the initial trigger for a similar cascade of events after repetitive mild TBI that includes dysregulation of microglial homeostatic functions and production of proinflammatory and neurotoxic substances that will then lead to neuronal damage (Figure 5). To better understand the mechanistic link between TBI and neurodegenerative diseases, developing new mouse models that allow us to investigate the factors involved in tau phosphorylation and aggregation is warranted. Characterizing the time-dependent cell-specific gene expression profile of glial cells and studying their involvement in pTau accumulation will also possibly open the doors to target the chronic inflammatory response after trauma and offer therapeutic intervention to limit the risk of dementia development in TBI patients. In addition, using more advanced clinical neuroimaging will be helpful to detect pathological tau deposition and neuroinflammation will be useful. Future longitudinal studies that take into consideration the age of onset, severity, repetitive nature of TBI and genetic background of patients and animal models are warranted to investigate the potential role of neuroinflammation as a link between TBI and long-term risk of worsening tauopathy and behavioral outcomes in adolescents.

## 4. Materials and Methods

### 4.1. Experimental Animals

Studies were performed using 38-day old (adolescent) male PS19 transgenic mice (P301S Tg mice) (Stock #008169, Jackson Laboratories, Bar Harbor, ME, USA) and C57/BL6J (Stock #000664, Jackson Laboratories). All procedures were performed in accordance with the NIH Guide for Care and Use of Laboratory Animals and followed protocols approved by the MGH Institutional Animal Care and Use Committee (Approval code: 2004N000286; Approval date: 08/20/2019). Mice had access to food and water ad libitum and were housed on a 12-h day-night cycle in laminar flow racks in a temperature-controlled room (25 °C). Investigators were blinded to study groups in all experiments. Mice were randomized to sham or rCHI at 38 (+/−3) days of age. Sham-injured and rCHI mice were housed in the same cage. Subsequent groups were injured at 38 (+/−3) days of age without any specific randomization scheme, as the mice were genetically identical and compared to shams.

### 4.2. Repetitive Closed Head Injury Model (rCHI)

A modified closed head injury (CHI) model was used as previously described [52]. Mice were anesthetized with 2.5% isoflurane in 70% N_2_O and 30% O_2_ for 90 s. Anesthetized mice were placed on a taught KimWipe napkin and grasped by the tail. The head was placed under a 42-inch long, 9/16-inch diameter brass guide tube. A 1/2-inch diameter 53 g lead cylindrical weight with a flat, unbuffered surface was dropped onto the dorsal aspect of the skull directly above the right (days 1, 3) or left (day 2) ear between the coronal and lambdoid sutures. After impact, mice were placed supine and loss of consciousness (LOC) time was recorded as the time to righting reflex. Sham-injured mice received anesthesia but no injury.

### 4.3. Preparation of Brain Tissue for Immunohistochemistry

Mice were deeply anesthetized with isoflurane and decapitated. The brains were removed and frozen in liquid nitrogen prior to sectioning. Sagittal sections (12 μm) were placed on Colorfrost Plus™ treated adhesion slides (Thermo Fisher Scientific, Waltham, MA, USA) using a cryostat. The brains were cut at 0.3 mm intervals from the medial to the lateral side, starting at 0.3 mm from the sagittal plane. For analyses using paraformaldehyde-fixed tissue, mice were transcardially perfused with PBS followed by 4% paraformaldehyde. Brains were post-fixed in 4% paraformaldehyde for 48 h, cryoprotected in 15% for 24 h, and then 30% sucrose, frozen at −80 °C, and cut on a cryostat.

### 4.4. CD11b Immunohistochemistry

PFA-fixed brain sections were washed twice with DPBS (without calcium and magnesium) before undergoing fixation with 95% ethanol (pre-chilled to 4 °C) for 10 min. Antigen retrieval was performed with a 15-min incubation of pre-warmed (37 °C) trypsin (0.25%) before blocking endogenous peroxidase activity with a 5-min incubation of 0.3% hydrogen peroxide and 0.3% normal rabbit serum in DPBS. Sections were blocked in DPBS with 1.5% normal rabbit serum for 20 min before primary antibody incubation with 1:100 rat anti-mouse CD11b (Bio-Rad MCA711G, Hercules, CA, USA) in DPBS with 2.5% normal rabbit serum. The primary antibody was visualized with the avidin-biotin horseradish peroxidase technique in accordance with the manufacturer’s instructions (Vector Laboratories, Vectastain Elite Kit PK6104, NovaRED Peroxidase (HRP) Substrate Kit SK-4800, Burlingame, CA, USA). Sections were then counterstained with hematoxylin in accordance with the manufacturer’s instructions (Vector Laboratories, H-3401, Burlingame, CA, USA), and mounted with Vectamount Permanent Mounting Medium (Vector Laboratories, H-5000, Burlingame, CA, USA). Brightfield images were subsequently obtained using a Zeiss Axio Scan.Z1 slide scanner with the ZenBlue software (Carl Zeiss, Jena, Germany).

### 4.5. AT-8 Immunohistochemistry

PFA-fixed brain sections were washed twice with DPBS (without calcium and magnesium) before undergoing fixation with 95% ethanol (pre-cooled to 4 °C) for 10 min. Antigen retrieval was performed with a 15-min incubation of pre-warmed (37 °C) trypsin (0.25%) before blocking endogenous peroxidase activity with a 10-min incubation in Bloxall endogenous peroxidase and alkaline phosphatase blocking solution (Vector Laboratories, SP-6000-100, Burlingame, CA, USA). Sections were blocked in Mouse-on-Mouse IgG Blocking Reagent (Mouse-on-Mouse Detection Kit, Vector Laboratories, BMK-2202, Burlingame, CA, USA) for one hour, followed by a 5-min incubation in Mouse-on-Mouse kit diluent. Sections were incubated with primary antibody AT8 (1:250) in Mouse-on-Mouse kit diluent (Vector MOM Kit) for 30 min, before being visualized with the avidin-biotin horseradish peroxidase technique in accordance with manufacturer’s instructions. Sections were then counterstained with hematoxylin in accordance with the manufacturer’s instructions (Vector Laboratories, H-3401, Burlingame, CA, USA), and mounted with Vectamount Permanent Mounting Medium (Vector Laboratories, H-5000, Burlingame, CA, USA). Brightfield images were subsequently obtained using a Zeiss Axio Scan.Z1 slide scanner with the ZenBlue software (Carl Zeiss, Jena, Germany). 

### 4.6. MC1 and PHF1 Immunohistochemistry

PFA-fixed brain sections were washed four times for five minutes each in TBS with 0.25% Triton X-100 before quenching endogenous peroxidase activity with 0.3% H202 diluted in 90% methanol for 30 min. Sections were then blocked in 5% milk diluted in TBS for one hour before incubating in the primary antibody of paired helical filaments (PHF1) overnight (PHF1, 1:1000, diluted in TBS with 5% milk) at 4 °C. The following day, sections were washed four times for 5 min each in TBS with 0.05% Triton X-100, and then incubated with secondary antibody (goat anti-mouse IgG1 1:1000) in 20% superblock with 0.05% Triton X-100 in TBS for two hours at room temperature. Four more washes were performed followed by an hour-long room temperature incubation in streptavidin HRP (1:1000) diluted in TBS with 0.05% Triton X-100 and 20% Superblock. After three TBS washes, the primary antibody was visualized with diaminobenzidine according to the manufacturer’s instructions (Vector Laboratories, SK-4100, Burlingame, CA, USA). We also stained for conformational tau (MC1, 1:100, diluted in TBS with 5% milk) using the protocol stated above. MC1 is a conformation-dependent antibody that reacts with both the N terminus (amino acids 7–9) and an amino acid sequence of tau in the third MTB (amino acids 313–322) that is necessary for in vitro formation of filamentous aggregates of tau similar to those seen in AD. PHF1 and MC1 Tau antibodies and protocols were generously provided by Dr. Peter Davies, The Feinstein Institute for Medical Research, Bronx, NY, USA.

### 4.7. GFAP Immunofluorescence Staining

PFA-fixed brain sections were washed three times with DPBS (without calcium and magnesium) before incubation in DPBS with 10% normal goat serum for 15 min. DPBS with 10% normal goat serum was used for overnight primary antibody incubation with GFAP (1:500) at 4 °C. The following day, brain sections were washed three times before a 90-min incubation at room temperature with secondary antibody (Alexa Fluor 488 goat anti-rabbit, 1:300, in 10% normal goat serum diluted in DPBS). Brain sections were then washed three times before nuclear counterstaining with DAPI.

### 4.8. Image Analysis

Analysis of percent CD11b, AT-8, and GFAP positive surface area was performed on 8–10 photomicrographs per animal (*n* = 4 for WT and P301S mice for both sham and rCHI conditions). The sections analyzed were taken between 300 and 1500 micrometers laterally from the sagittal plane. Each scanned photomicrograph was used to produce images from four different areas of the cortex and one image from the hippocampus. All the images were analyzed using ImageJ software (National Institute of Health, https://imagej.nih.gov/ij/). For CD11b and AT-8, images were split by color channel, and the channel of interest was thresholded using the Yen setting. For GFAP, images were split by color channel, and the channel of interest was thresholded using the default setting. Then, the percent surface area occupied by each staining was analyzed. In addition, we generated 500 × 500-micrometer square boxes in both cortical and hippocampal regions and performed manual quantification of CD11b positive cells for WT and P301S mice (*n* = 4 WT, *n* = 3 P301S for both sham and injury condition).

### 4.9. Western Blot

For brain tissue samples, cortex, hippocampus, and striatum were carefully dissected and frozen in liquid nitrogen. Brain tissue was homogenized in RIPA buffer (50 mM Tris-HCl pH 7.4, 150 mM NaCl, 1% NP-40, 0.25% deoxycholic acid, and 1mM EDTA: EMD Millipore, Burlington, MA, USA) with phosphatase and protease inhibitors (Thermo Fisher Scientific, USA). Samples were sonicated and centrifuged at 10,000× *g* for 30 min (Eppendorf 5424R; Fisher Scientific, USA) and the supernatant was collected. Protein concentration was measured using a DC™ protein assay (Bio-Rad, Richmond, CA, USA). Samples were denatured in buffer containing SDS and beta-mercaptoethanol and electrophoresed in precast 4–20% Tris-HCl gels (Bio-Rad, Richmond, CA, USA) and dry transferred onto polyvinylidene fluoride membranes using iBlot 2 (Thermo Fisher Scientific, Waltham, MA, USA). After blocking in 5% BSA for 1 h, membranes were incubated at 4 °C overnight with the primary antibodies: AT8 (Invitrogen, Waltham, MA, USA, 1:1000). Horseradish peroxidase-conjugated secondary antibody was used for ECL (EMD Millipore) detection. The results were normalized to β-actin (Cell Signaling Technology, Danvers, MA, USA, 1:5000). Densitometry was performed using image analysis (ImageJ).

### 4.10. Behavioral Studies

#### 4.10.1. Open Field Testing

Mice were individually placed in housing cages with clean bedding and covered by a thin wire grid. During the open field test, mice were recorded by ceiling-mounted cameras, and their movements were tracked by AnyMaze as described [52]. The recording lasted for 30 min and the distance covered during that time was used as a marker of overall activity.

#### 4.10.2. Plus Maze

Plus Maze was performed as previously described [52] and the percent of time spent in the center, closed and open arms were analyzed by Any Maze software.

#### 4.10.3. Rotarod

Mice were placed on a Rotarod apparatus (Harvard Apparatus, Holliston, MA, USA), accelerating from 4–40 RPM in 120 s. Trials began by placing the mouse on the rod and acceleration began. Each trial ended when the mouse fell off the rod, and the latency was manually recorded. Mice were tested for 5 trials a day (1-min inter-trial interval) for 4 consecutive days.

#### 4.10.4. Morris Water Maze

Morris Water Maze was performed as previously described [60] with 7 hidden and 2 visible platform trials and 90s maximum latency to the platform. The time until the mouse mounted the platform (escape latency) was measured and recorded (AnyMaze 8.42, Stoelting, Wood Dale, IL, USA). For probe trials, the platform was removed, and the time spent in the target quadrant (total 30 s swim time) was recorded.

### 4.11. Statistical Analysis

Data points are the mean ± standard error of the mean (SEM). Student *t*-test was used to analyze data for CD11b, AT8, and GFAP surface area quantifications. Western blot densitometry data were analyzed by Tukey’s multiple comparisons tests to compare WB/AT8 data of 4 different groups. MWM and Rotarod were analyzed using two-factor repeated measures of analysis of variance (ANOVA; group × time). Probe trial, elevated plus maze, and open field testing were analyzed by unpaired *t*-test. Statistical analyses were performed using GraphPad Prism 8 software (GraphPad Software Inc., La Jolla, CA, USA) and differences for all tests were considered significant if the *p*-value was <0.05.

## Data Availability

Data is contained within the article.
